# Importance of environmental policy on firm performance for the textile industry: A contextual study of Pakistan

**DOI:** 10.3389/fpsyg.2022.1008890

**Published:** 2022-11-30

**Authors:** Tahir Iqbal, Muhammad Asim Shahzad, María J. Alonso-Nuez, Jorge Rosell-Martínez

**Affiliations:** ^1^Department of Business Administration, University of Zaragoza, Zaragoza, Aragon, Spain; ^2^Superior College Lahore, Lahore, Punjab, Pakistan; ^3^Jiangsu University, Zhenjiang, China

**Keywords:** public participation, government regulations, organizational environment management system, environmental performance, environmentalism, organizational performance, textile sector, Pakistan

## Abstract

Global warming and air pollution are severe threats to humans and ecosystems. While some of these issues, particularly those on a small scale requiring low-cost behaviors, may be relieved by developing and implementing environmental policies, it is clear that legislative measures and behaviors requiring a significant degree of sacrifice are required. The goal of this research is to highlight the aspects that can contribute to improve organizational performance in Pakistan’s textile industry. Through the moderating function of environmentalism, the study examines the impact of public participation, government regulation, and organizational environmental management systems (EMSs) on environmental and organizational performance The findings show that public participation, government regulation, and the organizational EMS all have a significant impact on both environmental and organizational success. Data were collected *via* a survey questionnaire. Smart-PLS-3 was used to evaluate the data. Furthermore, if environmentalism is regarded as a moderator, the overall influence on organizational performance will be greater. Public participation, government regulations, and an organization’s EMS are all seen to have a substantial influence on both environmental and organizational success.

## Introduction

According to studies, increased public knowledge of environmental deterioration and increased fears about public health encourage residents to push governmental authority to affect environmental decisions. Despite the promises stated in the legislation’s language, Pakistani society lacks a significant institutional structure to encourage public engagement in environmental preservation. The Pakistani government reacts to public demands on an *ad hoc* basis, with little institutional commitment to addressing citizens on environmental matters. This is sad, as public policies that do not allow for meaningful public input are constantly tainted by exploitation.

The willingness of local governments to implement environmental rules and public engagement in environmental management varies greatly throughout Pakistan’s regions. Most inland regions with a poor economy are more prone to disregarding environmental regulations. Local politicians are often more interested with local economic growth than environmental conservation (Langpap and Shimshack, 2010).

Firms’ actions not only provide the intended outputs, but they also pollute the environment by generating wastewater, producing noise pollution, air pollution, and land pollution, all of which have a detrimental influence on our ecosystem. As public awareness of environmental contamination grows, the topic of environmental management becomes more significant.

The placement of polluting enterprises is also influenced by regional variances in environmental policies. According to the well-known pollution haven theory, polluting companies will seek to migrate to nations with less regulations, resulting in pollution havens. On the one hand, the Heckscher–Ohlin model is principally responsible for theoretical studies of pollution havens (Meesala and Paul, 2018). They developed a North–South general equilibrium model in their seminal work to explain the link between international commerce and pollution. The North, in their paradigm, is a more developed country with a better standard of living, whereas the South is a less developed country with a lower standard of living. Both countries control the environment through pollution charges. According to their findings, in a free-trade situation, the developed North will choose for a high-level environmental tax. As a result, all polluting businesses are forced to shift to the South, which has lower pollution taxes. Copeland and Taylor’s model was recently extended to include a broader framework, providing strong theoretical support for the pollution hypothesis (Taylor, 1992).

The majority of empirical research on pollution havens to date has been on the inter-country migration or intra-country relocation of polluting enterprises in developed economies such as the United States (Condliffe and Morgan, 2009). On the other hand, the influence of environmental rules on intra-country industrial mobility has received less study in developing economies such as Pakistan. Additionally, the bulk of studies concentrate only on enforcing official regulations, overlooking the critical role of public participation in environmental governance. Additionally, previous research has frequently used the cost of pollution abatement or the intensity of pollutant emissions as proxy metrics for environmental legislation. These proxies may pose a worry about endogeneity into regression analysis (Cole et al., 2013).

Avoiding pollution may enable firms to reduce energy consumption, regulate expenses, and reuse commodities through the recycling process (Greeno, 1992; Taylor, 1992; Hart, 1997; Rafiq et al., 2022).

In democratic ideals and good governance, the accountability for individuals and their right to freedom is the key characteristics of what is required. To effectively involve the public in decision-making processes, a government would begin by providing them with necessary information about the issues that they are concerned about, then provide venues for them to express their opinions and consider alternative viewpoints, and finally empower the public to make decisions that are in their individual and collective interests. Taking the form of a political ideal, public participation lays the groundwork for transparent administration. In addition to political philosophers, economists (Iraldo et al., 2009) have advocated for more public access to environmental information as well as greater public engagement in environmental decisions. An information-seeking residual claimant should be provided with information about the contract’s execution and should be empowered to make choices on the basis of such information, according to neo-institutionalism principles of access to information and decision-making in contractual partnerships. Governments enter into agreements with the people they represent with the goal of preserving the environment through the use of tax payers’ (public) funds. Since pollution has a negative effect on the health of the general people, they are the remaining claimant in such circumstances. In this instance, the general public is the residual claimant since pollution has a negative impact on their health, which is a type of residual expense for them (beyond the costs covered by polluters such as pollution fees, fines, factory closures, etc.). Because of this, citizens should be educated about the environmentally friendly enactment of the management and contaminators, and they should be empowered to make environmental decisions on their own behalf (Li et al., 2012).

## Literature review

### Public participation

The foundation of democratic values and effective administration are basis to respect the people and their right to self- determination. To involve the public in decision-making processes, a government must first provide the information that they need to understand the issues that they are concerned about, the public to voice their thoughts and evaluate alternative options then give the people the power to decide in ways that advance their own and the public’s interests. The political idea of public participation creates the foundation for open government (Ilyas and Rafiq, 2012). Ideas on public participation, which emerged in the 1960s in parallel with the introduction of ‘participatory democracy’ and were reflected by current perspectives on public involvement in several fields, including planning, by the late 1960s (Kathuria, 2007). Because of the potential benefits of exposing decision-making to a wide variety of public ideas, as interest in this topic expanded, the focus turned away from democratic involvement and toward deliberative participation. Jurgen Habermas has had a significant impact on this so-called “deliberative approach” (1984, 1987). Affirming Habermas’s claim that logical scientific, instrumental-technical thinking has supplanted moral and emotive-esthetic reasoning, we have seen a deterioration in the quality of our lives as well as our social, political, and economic lives.

As a way of eliminating the dominance of one type of thinking over others, Habermas’ communicative action theory supports fair, free, and open discussion and dialog among all participants. It is paradoxical that Habermas’ theories supporting adversarial forms of deliberation that reinforce the supremacy of instrumental-technical reasoning have led to the application of these concepts supporting adversarial forms of deliberation that reinforce the dominance of instrumental-technical reasoning. Take a look at this graphic to see what I’m talking about. There has been special interest in the potential for public contributions to enhance decision-making in the environmental sector, which has been particularly strong. In most cases, environmental problems are complicated, controversial, and well-researched issues.

Participation in environmental decision-making is viewed as a means of bringing a broader, more diverse range of knowledge and beliefs to bear on the complexities and ambiguity of these circumstances, as well as of reinforcing and maintaining the validity of the decisions that have already been reached. It is more likely that effective solutions will be found if public concerns and recommendations are taken into consideration and implemented. It also helps to build consensus and trust, which, in turn, helps to ensure that future problem-solving efforts will be financially viable in the long run. Additionally, well-established theoretical critiques as well as widespread contemporary concern that the scale and frequency of major problems are escalating, signaling a breakdown in established “rational decision-making” processes, have sparked this interest.

An example of this is the debate over genetically modified crops and foods, which has been used to show the limitations of knowledge and decision-making based on a restricted rational/ technical understanding of the issues at hand. BSE (Bovine Spongiform Encephalopathy) is an example of a congenital condition. The public’s confidence in institutions seen to be responsible for these worries, such as science, governments, and industry.

Official policy tools such as laws, regulations, and rules, as well as the pollution tax, are developed by the federal government; however, they are implemented by local governments. Furthermore, environmental control involves the participation of public actors, such as ordinary people, in a variety of ways (Simões et al., 2012). A growing number of studies suggest that raising public knowledge about environmental concerns has a positive impact on the environmental performance of polluting businesses in both developed and emerging countries, such as the United States and China.

### Organizational EMS

When the international organization for standardization (ISO) produced ISO 14001, an international standard on environmental management systems (EMSs), it was a watershed moment in the history of the world. The principal objectives of this standard are to enhance worldwide standards for environmental practices while also decreasing trade barriers through the reduction of taxes on imported goods. A sort of business practice and EMSs have an impact on both the environment and the performance of a firm (Rafiq et al., 2020). According to the new ISO 14001:2015 standard, an EMS is “a component of a management system that is used to manage environmental aspects, comply with regulatory requirements, and deal with risks and opportunities.” Operating procedures, products, and services provided by a firm that interact with the environment or have the capacity to do so are examples of environmental factors. EMSs are simply systems that identify environmental issues, determine their potential influence on the environment, assess their importance, and then provide the best feasible solution to minimize any negative environmental impact as a result of organizational actions, such as manufacturing. The ISO 14001:2015 standard now includes a new section on managing risks (threats) and opportunities (openings). Thus, prospective environmental hazards and opportunities for a firm are identified, as is the total effect of these hazards and chances. The best viable solution is then implemented to manage even minor concerns and capitalize on each opportunity. Acknowledging the needs and expectations of interested parties, as well as leadership commitment to an EMS, the establishment of an environmental policy and environmental objects, the determination of environmental aspects and their impacts and opportunities, the identification of environmental risks and opportunities, and the identification of the best possible solutions, all contribute to an organization’s overall environmental performance.

### EMS and environmental performance

One of the key objectives of an EMS is to improve the overall environmental performance of an organization. As stated by the international organization for standardization (ISO 14001:2015), “environmental performance” is defined as “performance related to the management of environmental factors” (Bennett and James, 1998). Any organization, regardless of age or size, may benefit from an EMS that is based on continual improvement in order to sustain environmental performance.

EMS stands for environmental management system, and it is a problem-solving and problem-identification tool based on the principle of continuous improvement. It may be used in a number of ways in an organization, depending on the sector of activity and the perceived needs of the management team.

In order to better understand, show, and enhance their environmental performance, many firms are attempting to figure out how they might do so. The environmental performance of an organization should be evaluated in relation to its environmental policy, objectives, targets, and other environmental performance criteria, among other things. In reality, an EMS is a well-organized and well-coordinated strategy for dealing with environmental problems in businesses in a proper manner, with the objective of enhancing their environmental performance. The measurable output of an organization’s environmental management (the results may be compared to the company’s environmental policy, environmental objectives, environmental targets, and other environmental performance standards, as well as other environmental performance standards). The results of an organization’s environmental management (the results may be compared to the organization’s environmental policy, objectives, and targets), and assessing environmental factors (components of an organization’s activities, commodities, or services that have the potential to interact with the environment) is essential in any case in order to evaluate an organization’s environmental performance. Environmental impacts are described as changes to the environment that are either negative or positive in nature and that are induced totally or partially by environmental variables. Environmental effects can be either negative or positive in nature. A cause-and-effect relationship exists between environmental conditions and the impact they have on human health. Moreover, an environmental component with a significant environmental effect is one that has had or has the potential to have a significant environmental impact. When developing, implementing, and maintaining an EMS, the organization must ensure that significant environmental aspects are taken into account. Identifying significant environmental aspects and their associated impacts is necessary to determine whether and where control or improvement is required, as well as to establish management action priorities. The establishment of comprehensive, appropriate for independent inspection, replicable, and verifiable significance criteria for the organization’s operations, goods, and services is required in order to determine the major environmental elements of the organization’s operations, goods, and services.

### EMS and firm performance

The financial performance of a firm is determined by the effectiveness of its EMS and improves the environmental performance of a company through the use of an EMS. When a company’s environmental performance is good, it not only reduces costs by generating less waste, but it also establishes differentiation, well-recognizability, a soft image, and brand reputation among customers, consumers, and legal authorities, all of which contribute to an improvement in the firm’s economic performance over time. When it comes to managing the link between the firm and the environment, the EMS is a good place to start. The purpose of an EMS is to improve the overall environmental performance of a company. Performance should be tracked and managed *via* the use of measurements and indicators. Essentially, indicators are variables that summarize or otherwise simplify crucial information about the state of a complicated system. For an effective assessment of environmental performance, the selection of acceptable “raw” data and the establishment of relationships between “raw” data are critical.

### Government regulation and environmental performance

Government regulations are an important part of environmental performance. If, the government is having intention to focus on environment, then moves toward environmental litigation and induce organizations to follow them. In developing nations, the tendency of developing environmental laws is under dearth. In addition, local governments in developing countries do not receive sufficient incentives to reduce environmental pollution, which is a problem in these countries. In fact, local governments have actively competed for input elements to create area economies and strict environmental regulations have also evolved into a policy to surpass economic growth (Wang et al., 2003; Li and Wu, 2017). This competition has resulted in an increase in the number of countries that have stricter environmental regulations (Deng et al., 2012; Lan et al., 2012). Local governments are not hesitant to adopt laxer environmental standards than those in neighboring districts in order to draw capital inflows, particularly foreign direct investment (FDI). In addition, the Porter hypothesis, which is a traditional economic theory, suggests that environmental legislation may encourage corporations to innovate more activities of businesses, thereby improving those businesses’ productivity as well as their environmental performance and their ability to compete. Despite this, there is still a lack of consensus regarding the presence of such an effect in China. Furthermore, the majority of related studies focus on examining the connection between governmental regulation and growth in (environmental) total factor productivity while taking into account total pollutant emissions in developing nations. However, no specific study has yet been done on how environmental regulation affects environmental performance while taking into account both carbon and air pollutant emissions.

### Public participation and environmental performance

The use of public engagement strategies to address environmental problems is one area in which non-governmental organizations (NGOs) have a history of improving through time. According to Thapa et al. (2013), nongovernmental organizations are the primary actors in promoting and campaigning for empowered participation. Hasana et al. (2018) evaluate public participation in EIA by comparing environmental projects led by governmental organizations and NGOs. They claim that NGO-led projects play a crucial role in ensuring participation in all stages of EIA, as well as smoothing stakeholders’ expectation conflicts. This research was published in Environmental Impact Assessment. Over the past few years, a multitude of researchers have carried out comparable work using data from China. Wu et al. (2018) investigate the impacts of public participation on environmental performance using panel data from 31 Chinese provinces over the period of 2004–2015. They claim that environmental petitions are significantly correlated with non-binding environmental pollutants. Their findings were published in Environmental Research Letters. According to the findings of Li L. et al. (2018), the publication of the China Air Pollution Map reduces the industrial pollution emission, but not in a consistent manner. This suggests that government policies, public participation, and enterprise involvement should all be involved in the process of resolving environmental issues. Li G. et al. (2018) use a difference-in-differences (DID) model to investigate the role of environmental non-governmental organizations (ENGOs) in China’s urban environmental governance model. They come to the conclusion that the influence of ENGOs is more prevalent in eastern and central China than in western China.

### Environmental performance and organizational performance

There have been a number of investigations into the relationship between environmental responsibility and organizational performance; nevertheless, the findings of these studies have been inconclusive (Jo et al., 2015). The findings of an investigation on the causal connection between a reduction in environmental pollution and organizational performance that was carried out by Hart and Ahuja (1996) revealed that there was no consensus regarding the existence of this causal linkage. In addition, a number of researchers have stated that substantial investments made by businesses in environmental management will result in increased costs and diminished benefits. As a consequence of this, it is essential that businesses evaluate the potential benefits against the costs associated with these investments (Palmer et al., 1995). On the other hand, other academics have pointed out that stricter environmental regulations might motivate businesses to invest in innovative production equipment in order to reduce environmental pollution and production expenses, which ultimately results in increased profitability (Lee et al., 2016). According to Hutchinson (1992), businesses need to rely on environmentally friendly activities in order to achieve possible benefits. Some of these benefits include an improved organizational reputation, attracting customers who are concerned about environmental pollution, reducing production expenses by conserving power, developing positive relationships with local communities, and producing environmentally friendly products. Businesses have the potential to increase their competitiveness by making early investments in environmentally friendly technology. This is due to the fact that environmentally sound technology is likely to result in lower unit costs of production as well as the development of successful organizational practices (Nehrt, 1996). In addition, regulations concerning the environment require businesses to upgrade their production technology and, as a result, improve their competitive advantages over a longer period of time. This is because environmentally friendly machinery has the potential to reduce costs as a result of increased production efficiency, which, in turn, enables businesses to achieve improved levels of competitive advantage (Porter, 1998).

## Research questions and methods

The purpose of this research is to address three difficulties and examine other concepts that are related to them. To begin, what drives individuals to become involved in environmental decision-making is unclear. People with an interest in environmental issues are more likely to participate in decision-making processes or to respond to decisions that have already been made when they have a stake in those decisions. Those who are not affected by a project could be against it because they care more about the ecological than the economic value of natural resources, but local residents may oppose it because they are concerned about their own livelihood. Those who are attached to an area through ownership, having a family, or having a career are more likely to be concerned about the project’s environmental and health effects and to participate more actively than those who are not tied to the location. Second, who are the individuals or organizations in charge of supporting public participation? In Pakistan, environmental protection agency employees are responsive to public environmental concerns and serve as important facilitators of public participation in environmental conservation efforts. The important are the contributions of ENGOs and other civic organizations, such as homeowner associations (HOAs). And last, to what degree have the laws made it simpler for citizens to take part in the political process? The legislation establishes a legislative context that supports public involvement in environmental protection and provides legal support for their arguments. As a result, they create an environment that is favorable to environmental action. In order to select instances for the study from among the many environmental issues that exist in Pakistan, we used the following three criteria: (1) public engagement; (2) government restrictions at three levels: community, regional, and national; and (3) occurred in areas with varying levels of economic growth. As a result, we picked three cases of environmental campaigning that were well-known across the country for the research. With the vast range of geographic scopes and administrative levels available, it is possible to have a thorough picture of why various groups of people are worried about different environmental concerns, as well as the conditions that motivated them to take action in the first place. Furthermore, the geographic and socioeconomic inequalities that exist between individuals allow for the investigation of variances in organizational strategies to be conducted. The information for the three instances was gathered from different academic sources both private and public sources. Data analysis was done through the use of analytical narratives and the approach of comparative case studies. We are able to move past attempts to define a globalized, harmonious society and to connect with “local knowledge’s,” or components of experience that are specific to the actors and case contexts and tell us something crucial about the driving forces behind certain claims as well as social interactions, by recovering the stories of how the cases have developed over time (Bennett and James, 1998). Using the case study approach, you would do so because you consciously wanted to cover contextual elements, and you assumed that they may be enormously relevant to the phenomena under examination,” the author writes (Yin, 2003, p. 13). If we discover that the drivers and agents differ depending on the context by comparing the three cases, the findings would be a promising first step toward theoretical replication that significantly improved when compared to findings. The next section includes a summary of the study objectives and procedures, followed by the accounts of three environmental activism occurrences that occurred in Pakistan.

In light of this, the purpose of this article is to investigate the link between drivers (government regulations, environmental performance, and organizational performance), government regulation, and performance (environmental and organizational performance). The relevant constructs were measured using a survey-based technique, and the hypothesized correlations were tested using structural equation modeling. From three perspectives, this work will add to important research. First, this research elucidates the unique roles of EMSs, government laws, and public engagement in the execution of environmental policy, enhancing knowledge of how to improve organizational and environmental performance. Second, using empirical testing on a large sample dataset, this study reveals a substantial link between public engagement, government rules, organizational EMS, and environmental and organizational performance. The findings support the efficacy of environmental policies. Third, this research focuses on the Pakistani textile sector in the context of a rising economy. Pakistan’s textile industry is now undergoing fast growth.

## Methodology

The research approach is determined by the study’s goal and problem (Hillary, 2004) and appropriate approaches are required for accurate results. The quantitative strategy to research was chosen after assessing the problem and purpose of the current study, and the data were collected using a cross-sectional method. The researchers utilized a questionnaire to gather data for this study. For the current research project, it is desirable to utilize a survey questionnaire since it enables data collection in a reasonable period of time and is a financially advantageous data collection method (Richter et al., 2016). Furthermore, this strategy ensures respondent confidentiality and allows sensitive data to be easily obtained. The scale items were adapted from previous research.

The technique includes information on the study design, population, sampling, composition, questionnaire reliability, and data collecting, as well as valuation processes. The findings of the construction information were determined by analyzing the obtained data. The quantitative technique, which is based on primary data, was employed since it is a tool for studying and investigating the subject matter for research. The population in this study is made up of lower to upper-level employees who work in Pakistan’s textile industry. The data were collected using a technique called convenient sampling. A total of 500 questionnaires were issued, with 460 being collected from responders. Out of 500 questions, 57 are unconfined, making it impossible to analyze the data. The remaining 403 questionnaires are subjected to statistical analysis. The surveys were designed using an adaptive technique, with a structured questionnaire based on a seven-point Likert scale, with “7” representing robust agreement and “1” representing robust disagreement with the topic. The questions were chosen based on previous high reliability in Pakistan and other nations. Table 1 lists the questions and their references.

**Table 1:** Table of construct.

Sr. No	Construct	Items	References
1	Public participation	3	Langpap and Shimshack (2010)
2	Govt. regulation	5	Kathuria (2007)
3	Organizational EMS	4	Dao et al. (2011)
4	Environmentalism	5	Hillary (2004)
5	Environmental performance	5	Eccles et al. (2014)
6	Organizational performance	4	Rawashdeh (2018)

We have been used a causal melding approach and descriptive analysis by Smart PLS (Partial Least Squares).

## Results

### Descriptive analysis

**Figure 1:**
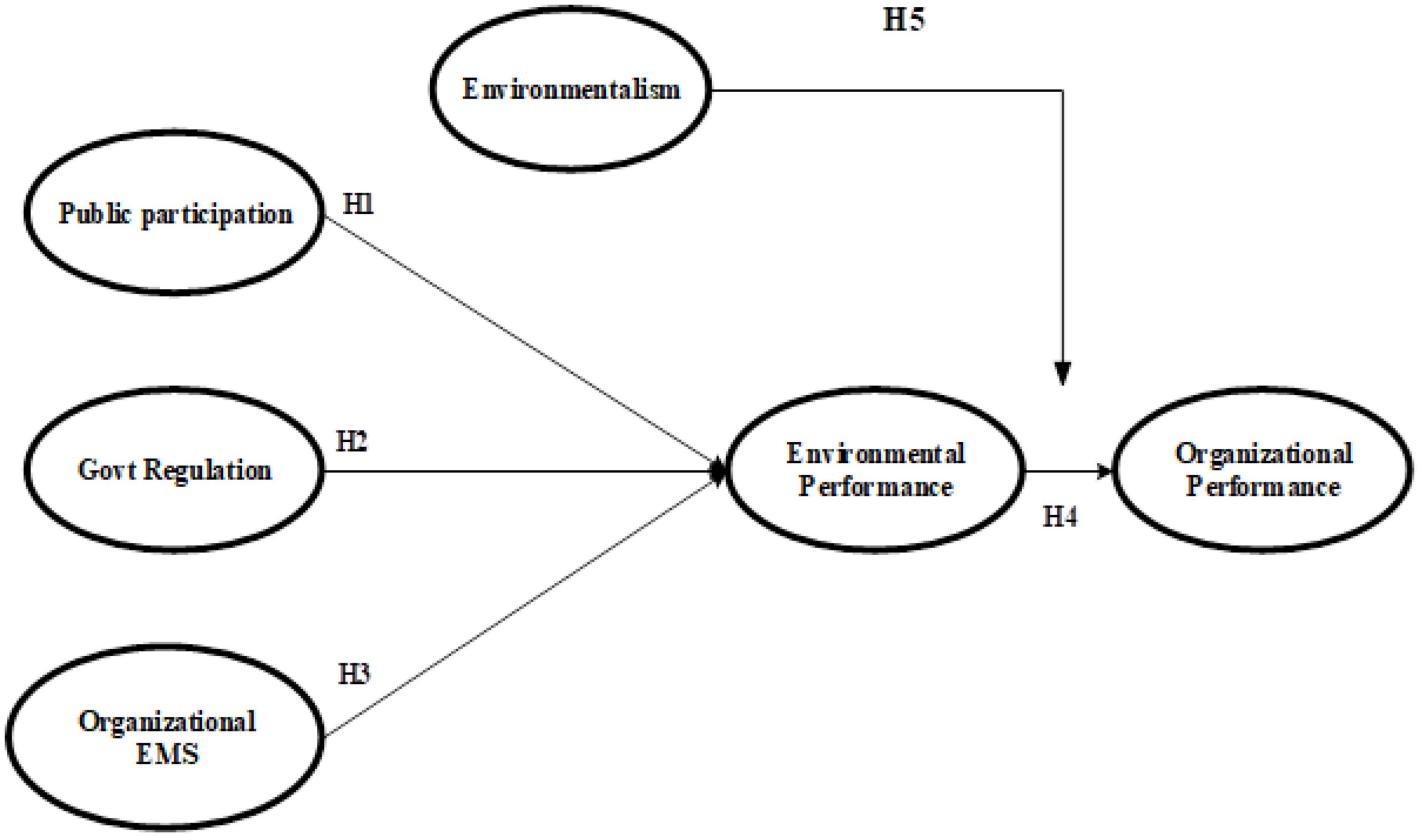
Theoretical framework.

A total of 500 questionnaires have been issued. Four hundred and sixty questionnaires were gathered from respondents, and 57 surveys were left unbound, making it impossible to analyze the data. As a result, we submitted 403 questionnaires into Smart Pls 3 to conduct descriptive analysis and examined demographic features of textile industry respondents. Gender, age, education, designation, and experience are all demographic factors in this study (Figure 1).

*Hypothesis (H1)*: Public participation has a positive impact on environmental performance and organizational performance in textile sector of Pakistan.

*Hypothesis (H2)*: Govt regulations have a positive impact on organizational performance and EP implementation in textile sector.

*Hypothesis (H3)*: Organizational EMS in TEXTILE SECTORE has a positive impact on environmental performance and organizational performance.

*Hypothesis (H4)*: Environmental performance has a positive impact on organizational performance.

*Hypothesis (H5)*: Environmentalism positively strengthens the relationship between environmental performance and organizational performance.

### Analysis and results

This study used Smart PLS 3 (SEM) for data analysis and used a two-step process to provide analytical results (Gliem and Gliem, 2003).

### Measurement model assessment

**Figure 2:**
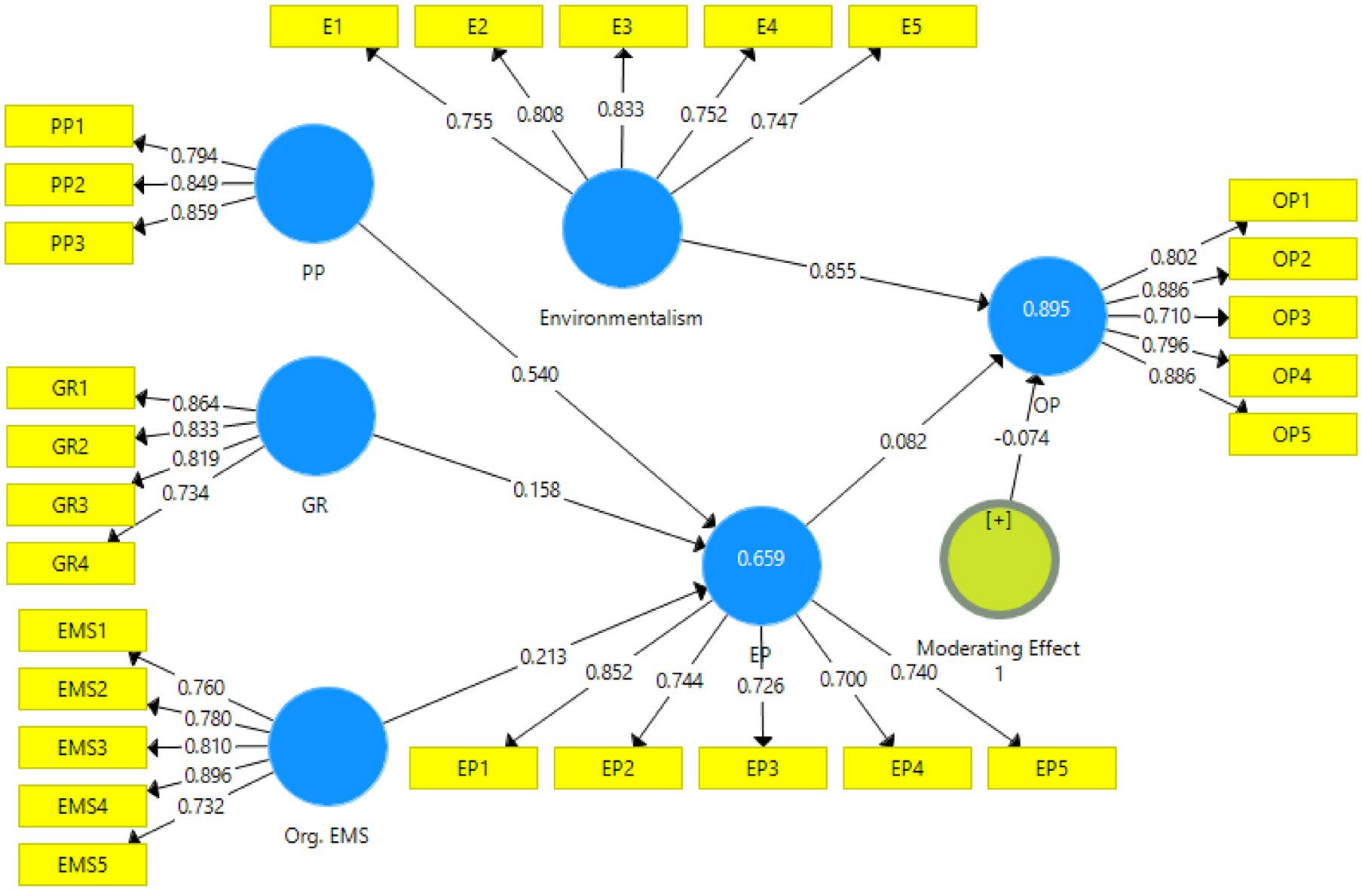
PLS algorithm.

**Figure 3:**
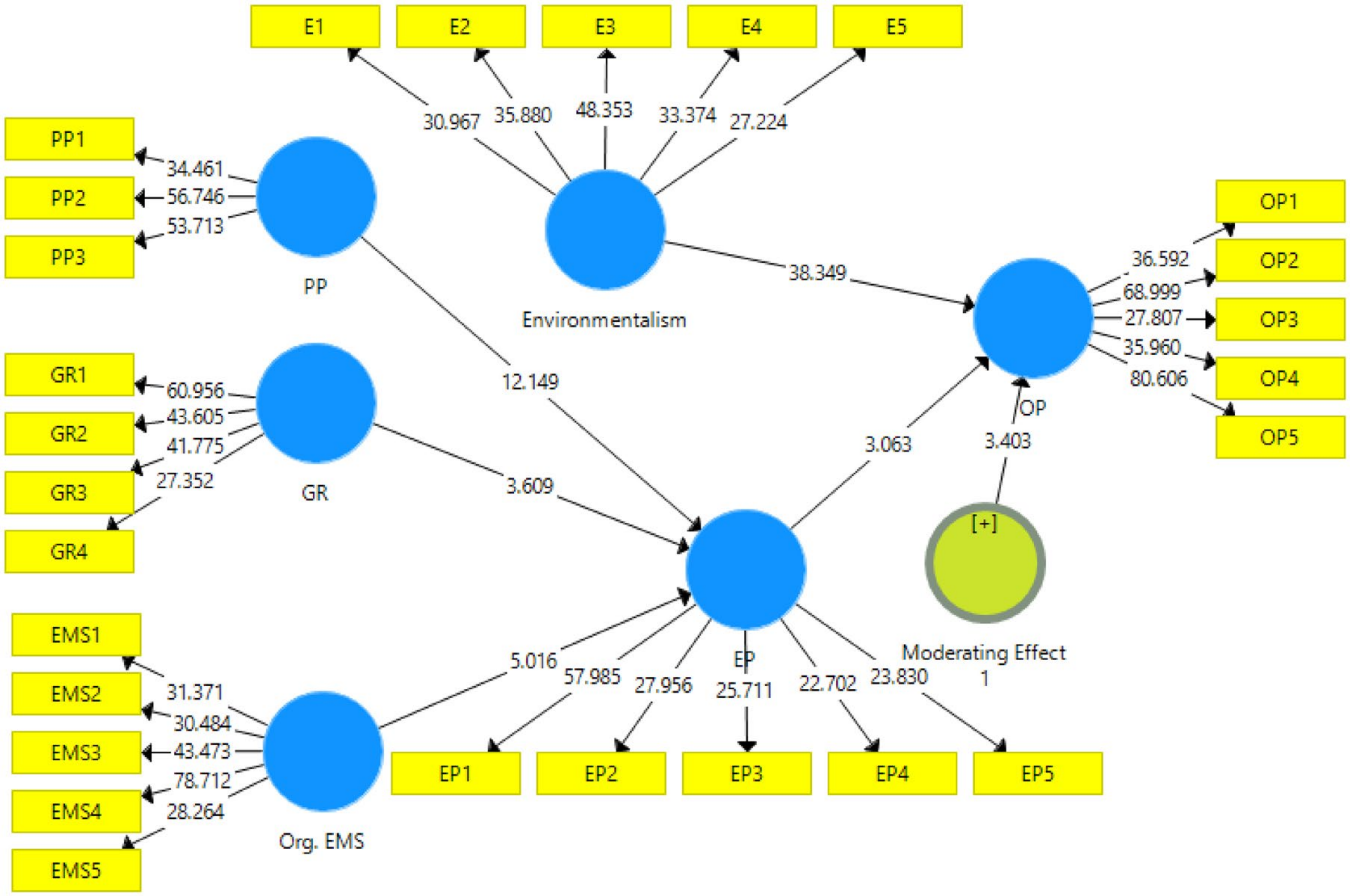
PLS bootstrapping.

The PLS-SEM measurement model was employed to examine the data’s reliability and validity (Serda, 2013). Factor loading, Cronbach’s alpha, composite reliability, and average extracted variance were used to assess construct reliability (AVE). The measuring model was also used to assess discriminant validity. The findings of the measurement model are shown in Figures 2, 3 and Table 2.

**Table 2:** Evaluation of the measurement model.

Variables	Items	FL	AVE	CR	Cronbach’s alpha
PP					
	PP1	0.794	0.697	0.873	0.781
	PP2	0.849			
	PP3	0.859			
					
GR	GR1	0.864		0.887	0.829
	GR2	0.833			
	GR3	0.819			
	GR4	0.734			
					
OEMS	OEMS1	0.760	0.636	0.897	0.856
	OEMS2	0.780			
	OEMS3	0.810			
	OEMS4	0.896			
	OEMS5	0.732			
					
Environmentalism	E1	0.755	0.608	0.886	0.839
	E2	0.808			
	E3	0.833			
	E4	0.752			
	E5	0.747			
					
EP	EP1	0.852	0.569	0.868	0.809
	EP2	0.744			
	EP3	0.726			
	EP4	0.700			
	EP5	0.740			
					
OP	OP1	0.802	0.670	0.910	0.875
	OP2	0.886			
	OP3	0.710			
	OP4	0.796			
	OP5	0.886			

The values of convergent validity of variables are shown in Table 2. The values of alpha’s value, composite reliability, and AVE may be measured according to the directions of convergent validity (Hsu, 2008). Because all of the numbers in this study are over the threshold levels, all of the variables have convergent validity. The threshold value for Cronbach’s alpha is 0.6 or greater than that. Additionally, the AVE value should be greater than 0.5, and the composite reliability value should be greater than 0.7 (Table 3).

**Table 3:** Structural model assessment.

	Original sample (O)	Sample mean (M)	Standard deviation (SD)	*T* statistics (|O/SD|)	*p*-Values
EP - > OP	0.082	0.084	0.026	3.132	**0.002**
Environmentalism	0.855	0.856	0.022	38.184	**0.000**
GR - > EP	0.158	0.159	0.043	3.645	**0.000**
Environmentalism - > OP	−0.074	−0.076	0.023	3.347	**0.001**
OEMS - > EP	0.213	0.212	0.041	5.177	**0.000**
PP - > EP	0.540	0.541	0.041	13.202	**0.000**

Mean, SD, *T*-values, and *p*-values. The bold value means satisfying the standard value.

### Structural model assessment

SEM-PLS structural model analysis was used to estimate the study’s hypotheses. According to the results of structural model research, public participation has a significant effect on company performance (β = 0.540, *t* = 13.202). As a consequence, the findings of the analysis support H1. Furthermore, this analysis found that government restrictions have a substantial positive association with company performance (β = 0.158, *t* = 3.645), and H2 is statistically acceptable (Table 4).

**Table 4:** Discriminant validity at construct level.

	EP	Environmentalism	GR	E* OP	OP	Org. EMS	PP
EP	**0.754**						
Environmentalism	0.793	**0.780**					
GR	0.645	0.726	**0.841**				
E* OP	−0.306	−0.193	−0.204	**1.00**			
OP	0.790	0.939	0.696	−0.286	**0.819**		
Org. EMS	0.655	0.751	0.659	−0.107	0.712	**0.798**	
PP	0.774	0.706	0.642	−0.239	0.724	0.626	**0.835**

The bold value means satisfying the standard value.

Table 5 shows the value of the square root of AVE, which is used to evaluate construct discriminant validity. The value of AVE square root should be higher than the value of other variables to obtain discriminant validity (Eccles et al., 2014).

**Table 5:** Structural model assessment (direct relation effect and results).

Hypotheses	Relationship	Beta	SD	*T* statistics	*p*-Values
H1	PP - > OP	0.540	0.041	13.202	0.000
H2	GR - > OP	0.158	0.043	3.645	0.000
H3	EMS - > OP	0.213	0.041	5.177	0.000

Source: Estimates made by the authors using data.

The bootstrapping approach is used to estimate the mediation effect using PLS-SEM. The results of the research showed that corporation completely mediates the relationship between environmentalism and organizational performance (β = 0.147, *t* = 4.567) and supported H4 (Table 6).

**Table 6:** Structural model assessment (indirect mediation).

Hypotheses	Relationship	Beta	SD	*T* statistics	*p*-Values
H4	PP- > GR - > OP	0.082	0.026	3.132	0.002

Source: Estimates made by the authors using data.

Table 7 summarizes the results of the moderation analysis. The data demonstrated that environmentalism had a significant and favorable moderating influence on the connection between environmental performance and organizational performance (β = −0.074, *t* = 3.347), supporting H5 (Table 8).

**Table 7:** Structural model assessment (moderation effects).

Hypotheses	Relationship	Beta	SD	*T* statistics	*p*-Values
H5	EP*E - > OP	−0.074	0.023	3.347	0.001

Source: Estimates made by the authors using data.

**Table 8:** R^2^ Value of endogenous structural.

Predictor construct	Target construct	R^2^	R^2^ adjusted	Predictive accuracy
PP, GR, OEMS, EP, and E	EP	0.659	0.656	Substantial
	OP	0.895	0.894	Substantial

## Discussion and conclusion

The goal of this research is to look into the function of public participation, government regulations, and organizational EMS in organizational success, with the environmental performance as a mediating factor. The goal of this study was to look at the moderated mediation impact of environmental performance, as well as the function of environmentalism as a moderating factor. According to the conclusions of the investigation, public engagement, government regulations, and OEMS have a favorable and substantial relationship with environmental performance. Public engagement, government regulation, and OEMS, on the other hand, are sector-specific and confirm a long-term economic edge in a competitive context. Furthermore, the findings of the study demonstrated that ENVIRONMENTAL PERFORMANCE has a crucial influence in the relationship between public engagement, government laws, and OEMS and organizational performance (Li, 1998). Companies that are more sustainable, according to research, excel in terms of environmental performance, both in the short and long term. Environmentalists strongly alter the association between public engagement, government regulation, and OEMS and organizational performance, according to the findings.

From a practical standpoint, the findings of a recent study have a number of consequences for senior management in the textile industry. A healthy environment is critical for a company’s success, and environmentalism is one of the most significant components that boosts the beneficial impact of environmental performance on company performance. For improved performance in a competitive market, top management of companies must set environmental policies and build a knowledge-sharing atmosphere inside the company. Firms can also increase their performance by focusing on environmental sustainability.

This study has a number of flaws that need to be studied in the future. The study’s sample was initially restricted to those working in Pakistan’s small- and medium-sized textile industries that make it challenging to extrapolate the findings to new businesses or production facilities. Second, this study only looked at a tiny group of people from a certain region of Pakistan, ignoring the rest of the country.

The literature lists various other types of public participation and government regulations besides public participation and govt regulations that are both revolutionary and transformational in relation to public participation, government regulations, and OEMS selected to represent the effect of these on environmental performance and organizational performance. Furthermore, the study concentrated on the influence of public engagement, government legislation, and OEMS on a particular environmental sustainability practice. Likewise, public engagement may not be the main determinant of environmental stewardship and corporate effectiveness. Other factors may play a role in interpreting this link, and they should be taken into account. Finally, the primary method of data collecting was quantitative, which may be viewed as a study restriction. In order to achieve the goals of the study more qualitative techniques should be used to gather more accurate data and findings as questionnaires and other self-reporting data collection processes may cause bias in responses. Finally, because this study was conducted in the context of Pakistani culture, its conclusions might only be applicable to Pakistani workers’ values, ethics, and beliefs in the workplace.

To accurately identify developing businesses, future research should replicate our findings across a sample of different businesses. Furthermore, a study should aim to collect samples from different locations across the nation in order to strengthen the generalizations made in the previous studies. It would also be interesting to check if the results matched or diverged from other studies if the researcher measured the various study components using various dimensions. Future studies on this connection should work to increase discernment by assessing novel traits in addition to leadership philosophies or that might affect how future leaders behave in organizations and other systems. Finally, applying mediating and moderating links to the literature and offering more clarification should be the main goals of future research. To obtain data that accurately depicts the study’s variables, researchers should use both quantitative and qualitative data collection techniques. Future researchers should employ structural equation modeling as an analytical strategy because it is thought to be the most effective at hastening the development of the study’s core model.

## Data availability statement

The original contributions presented in the study are included in the article/supplementary material, further inquiries can be directed to the corresponding author.

## Author contributions

TI collected and analyzed the data, and had a major contribution in this article. MS, MA-N, and JR-M reviewed the article and contributed to conclusion and provided significant suggestions. All authors contributed to the article and approved the submitted version.

## Conflict of interest

The authors declare that the research was conducted in the absence of any commercial or financial relationships that could be construed as a potential conflict of interest.

## Publisher’s note

All claims expressed in this article are solely those of the authors and do not necessarily represent those of their affiliated organizations, or those of the publisher, the editors and the reviewers. Any product that may be evaluated in this article, or claim that may be made by its manufacturer, is not guaranteed or endorsed by the publisher.
